# A school-based comprehensive lifestyle intervention among chinese kids against obesity (CLICK-Obesity): rationale, design and methodology of a randomized controlled trial in Nanjing city, China

**DOI:** 10.1186/1471-2458-12-316

**Published:** 2012-06-15

**Authors:** Fei Xu, Robert S Ware, Lap Ah Tse, Zhiyong Wang, Xin Hong, Aiju Song, Jiequan Li, Youfa Wang

**Affiliations:** 1 Department of Non-communicable Disease Prevention, Nanjing Municipal Center for Disease Control and Prevention, 2, Zizhulin, Nanjing, 210003, Peoples Republic of China; 2 Department of Epidemiology and Biostatistics, School of Public Health, Nanjing Medical University, Nanjing, China; 3 School of Population Health, The University of Queensland, Brisbane, Australia; 4 School of Public Health and Primary Care, The Chinese University of Hong Kong, Hong Kong, China; 5 Nanjing Jianye District Center for Disease Control and Prevention, Nanjing, China; 6 Johns Hopkins Global Center for Childhood Obesity, Department of International Health, Bloomberg School of Public Health, Johns Hopkins University, Room E2546, 615 North Wolfe Street, Baltimore, MD, 21205, USA

## Abstract

**Background:**

The prevalence of childhood obesity among adolescents has been rapidly rising in Mainland China in recent decades, especially in urban and rich areas. There is an urgent need to develop effective interventions to prevent childhood obesity. Limited data regarding adolescent overweight prevention in China are available. Thus, we developed a school-based intervention with the aim of reducing excess body weight in children. This report described the study design.

**Methods/design:**

We designed a cluster randomized controlled trial in 8 randomly selected urban primary schools between May 2010 and December 2013. Each school was randomly assigned to either the intervention or control group (four schools in each group). Participants were the 4^th^ graders in each participating school. The multi-component program was implemented within the intervention group, while students in the control group followed their usual health and physical education curriculum with no additional intervention program. The intervention consisted of four components: a) classroom curriculum, (including physical education and healthy diet education), b) school environment support, c) family involvement, and d) fun programs/events. The primary study outcome was body composition, and secondary outcomes were behaviour and behavioural determinants.

**Discussion:**

The intervention was designed with due consideration of Chinese cultural and familial tradition, social convention, and current primary education and exam system in Mainland China. We did our best to gain good support from educational authorities, school administrators, teachers and parents, and to integrate intervention components into schools’ regular academic programs. The results of and lesson learned from this study will help guide future school-based childhood obesity prevention programs in Mainland China.

**Trial registration:**

Registration number: ChiCTR-ERC-11001819

## Background

Childhood obesity has become a global epidemic [1]. China is one of many countries to be dramatically affected by this growing epidemic in recent years [2,3]. Obesity has many serious health and financial consequences [4-6]. Excess body weight during adolescence not only predicts adult obesity but also influences morbidity and mortality in adult life [7]. Treating obesity is both difficult and costly [8]. The obesity epidemic demands urgent population-based research on childhood obesity prevention programs to determine which programmes are effective and sustainable.

Schools are critical settings for promoting lifelong healthy eating and physical activity (HEPA) [9,10], and for preventing childhood obesity [10,11]. Several systematic reviews [12-15] and some recent studies [16-19] have shown that school-based prevention of obesity is feasible and effective; however other studies have not shown significant effects [20-22]. Although these school-based intervention studies have generally focused on improving healthy eating behavior and the promotion of physical activity (PA), different strategic components and follow-up periods make it difficult to compare the study results. Moreover, most of these studies were conducted in Western countries, and results from Western countries may not be directly applicable to the Chinese population, given obvious differences in school activities and structures between Western and Chinese societies (e.g., Chinese school students spend much more time in academic study) [23]. Hence, there is a need for a well-designed school-based intervention programme for obesity prevention in Chinese population.

To date, only four studies have investigated obesity prevention among school children in Mainland China, three in the language of Chinese and one in English [24-27]. These studies implemented individualized interventions focusing on controlling body weight in obese children. There is a lack of evidence concerning the effectiveness of school-based obesity prevention programs targeting overall students in China. We designed a cluster randomized controlled trial (C-RCT) to evaluate the effectiveness of a school-based comprehensive lifestyle intervention among Chinese kids against obesity (CLICK-Obesity) in 8 urban primary schools in Nanjing City, China. When designing the C-RCT we considered the unique cultural, academic and traditional aspects of Mainland China, as well as the urgent need for this type of intervention. This report presents the methodology used in this C-RCT.

## Methods

### Study design and recruitment of participants

**Figure 1:**
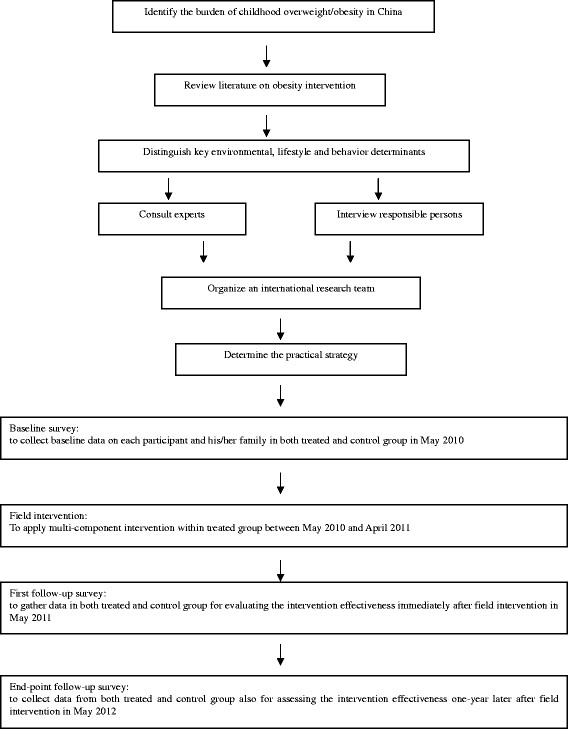
The Study Mapping Process of CLICK-Obesity Program.

This school-based C-RCT has being conducted from May 2010 to December 2013 in Nanjing, a large city in eastern China which had a registered population of more than 6.2 million in 2009. The intervention took place within Jianye, an urban district of Nanjing. In 2009, there were 13 primary schools in Jianye district, among which eight schools were randomly selected as target schools and randomly assigned to either the control or intervention group (four schools in each group). All the fourth graders in each of the selected schools were invited to participate in the study. Written informed consent was obtained from both the schools and parents/students’ guardians. Figure 1 presents the study mapping process. Although the entire project was designed to be three and half a year, the field intervention was planned to be implemented among intervention group within one academic year from May 2010 to April 2011, while three surveys were scheduled to collect data from all participants (both intervention and control groups) in this study: (1) The first survey was conducted in May 2010 to collect baseline data; (2) The second was in May 2011 to gather data for evaluating the intervention effectiveness immediately after field intervention; (3) The third will be in May 2012 to collect data also for assessing the intervention effectiveness one-year later after field intervention. This study was approved by the academic and ethical committee of Nanjing Municipal Center for Disease Control and Prevention.

### The intervention

#### The theoretical basis

The intervention component of our CLICK-Obesity study was developed based on the HEALTH-KIDS Study conducted by one of our team members in Chicago [17], but related changes were made to adapt to the situation in Nanjing in China. The HEALTH-KIDS intervention was designed based on several behavioral theories including the Theory of Triadic Influence (TTI) [28] and the Comprehensive School Health Program Model (CSHPM) by the United States of America Center for Disease Control (US CDC) [29], Briefly, the TTI theory was intended to account for factors that had direct and indirect effects on behavior and both new behaviors and regular behaviors, while the CSHPM model, a strategy designed for school-based behavior intervention projects, was recommended by the US CDC for school-based intervention programs to promote life-long healthy eating and physical activity habits.

#### Intervention components and implementation

**Figure 2:**
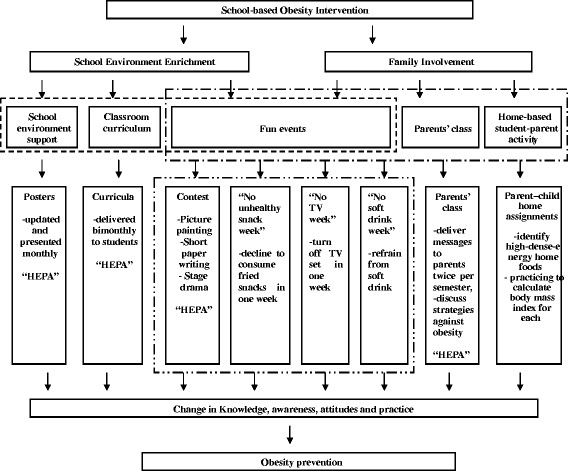
The Field Intervention Framework of CLICK-Obesity Program.

Students attending schools allocated to the intervention group received the field intervention program between May 2010 and April 2011, while students in the control group followed their usual health and physical education curriculum. As shown in Figure 2, the intervention consists of four components: a) classroom curriculum (including physical education and healthy diet education), b) school environment support, c) family involvement, and d) fun programs/events. The intervention was developed after taking full consideration of Chinese cultural and familial tradition, social convention, and the current primary education and examination system. In China, familial expectations usually lead to Chinese students being encouraged to spend a relatively large amount of time in academic studies, and to achieve highly competitive exam scores, and schools primarily define their quality and reputation by their students’ academic performance. These cultural and social contexts must be considered when designing and implementing any school-based lifestyle and behavioral intervention project in Mainland China.

All developed intervention components were integrated into the regular academic schedule of each school. For example, all schools in Mainland China are required by the educational authority to provide health-related curriculum each semester; we encouraged the intervention schools to use our curricula to meet this requirement (which all schools in the intervention group did). Another example is that all schools in China have a regular class session where parents/guardians are invited to attend. We encouraged the intervention schools to use these sessions (parent class) to provide parents/guardians with healthy diet and active lifestyle messages to prevent obesity among children and advice to help their children eat healthy food and engage in more physical activity after school.

a) Classroom curriculumThe classroom curriculum was designed to disseminate knowledge and skills to promote individual healthy eating behaviors and to increase inside-/outside-school physical activity. One 30-minute lesson was delivered by regular classroom teachers each month for one academic year (8 months) for the 4^th^ graders in the intervention group. All teachers were trained and provided with the same teaching materials to present for students. Members from our program team attended each class and sat in the back of the classroom to view each curriculum presentation lesson.The curriculum consists of two education modules: one focused on nutrition and the other on physical activity. The nutrition module aimed to help students reduce unnecessarily excessive energy intake, while the physical activity module focused on increasing energy expenditure. The nutrition module included an introduction to types of foods, and energy contained in different foods and practical advice on how to eat healthily on a daily basis. Students were shown what high-dense-energy foods are, for example Western snacks, soft-drinks and deep-fried foods. They were encouraged to consume low-dense-energy foods, such as vegetables and tofu. They were also offered tips on how to eat healthily, e.g., chewing thoroughly, and reducing fat intake by consuming meat without skin. The physical activity module encouraged students to engage in sufficient physical activity and to reduce screen-time. Students were also encouraged to do exercises inside or outside of the classroom during recess, and to walk to and from school.

b) School environmental supportSchool environment can be critical to helping students make desirable health-related behavioral changes and maintain a healthy lifestyle. Brief health-related messages and posters were presented in all intervention schools, in locations such as inside the classroom, gymnasium, playground, and cafeteria. The messages and posters were updated monthly according to scheduled intervention themes. Furthermore, posters made by students were posted on rear blackboards in classrooms in intervention schools. The posters promoted healthy eating and physically active behaviors. Selected examples of the blackboard poster titles included: “To be physically active is a good and wise way to reject obesity”, “Healthy eating can keep obesity away” and “Indulging in foods can guarantee your bad health”. Posters were updated by students monthly.

c) Family involvementParents/guardians are the gatekeepers for their children’s food choices, and they also serve as role models for their children by practicing and reinforcing healthy lifestyle choices [17,30]. Family involvement was developed to introduce families to the CLICK-Obesity intervention and to assist them to create a supportive environment for helping children change unhealthy behaviors and increase healthy eating and physical activity. The family involvement component included parents/guardians health class at school, in which they were invited to participate in an educational program twice per semester to learn appropriate strategies to advance healthy lifestyle choices against obesity. Families were given the opportunity to discuss practical aspects of the CLICK-Obesity program and to extend their knowledge on healthy lifestyle and eating behaviors. The curriculum designed for parents/guardians were organized by class teachers but presented by our team members each time, unlike the class curriculum which was presented to students by classroom teachers. After school family events were also offered, these included parent–child interactive home assignments, such as identifying high-dense-energy home foods, and practicing to measure body weight and height and to calculate body mass index.

d) Fun programs/events

(i) Presentation competition. Three competitions (picture painting, short paper writing and stage drama) were held in two semesters regarding (a) obesity and its associated risk to health, and (b) healthy lifestyle and behaviors against obesity in daily life. Students were encouraged to tell their own stories about healthy diet and physical activity behaviors. Picture painting, focusing on healthy lifestyle choices, and paper writing activities, focusing on healthy body weight, were implemented during the first semester. A stage drama, presenting how to keep a healthy body weight through healthy lifestyle and behaviors in daily life, was required to be presented by a classmate-team (at least two members) in the second semester. Six winners from each class were selected for each picture painting and paper writing competition, while one winner in each class was selected for stage drama show by a 7-member assessment panel, which including external experts (from the local Center for Disease Control and students’ health-care institute), teachers and classmates.

(ii) ‘No unhealthy snack week’. Students were encouraged to consume no high-dense-energy snacks for a week in early March 2011, and to take regular meals and fruits at home and school. A specific card designed to reinforce positive attitude and healthy practice was delivered to each participant in order to record his/her weekly snack consumption.

(iii) ‘No TV week’. In a selected week in late March 2011, we designed a program to encourage students to turn off TV at home and reduce computer-use to a minimal level at home and school. The parents/guardians received leaflets encouraging them to help their children do so at home. Students were also asked to report their daily screen-time during the week.

(iv) ‘No soft drink week’. In a week in late April 2010, students were advised to refrain from consuming soft-drinks and to drink pure fresh water instead. They were also required to record their soft drink consumption during that week.

### Data collection and measurement of key study variables

Anthropometric measures and interviewer-assisted questionnaire surveys were conducted at baseline (May, 2010), the first follow-up (May, 2011) and will be at the end-point follow-up (May, 2012). A structured 39-item questionnaire was designed to collect information on students' and their parents' socio-demographic characteristics, knowledge regarding obesity and its risk factors, intake of meat and vegetables/fruits, consumption of high-dense-energy snacks and soft-drinks, and physical activity. Additional information on lifestyle/behavior change was collected at the first follow-up survey. As part of the baseline survey parents/guardians completed a short questionnaire focusing on their families socio-demographic characteristics. Information collected included parental educational attainment, and family size and structure.

Trained Nanjing CDC research team members, each of whom had a university degree in a health-related discipline, conducted the physical examination and the survey in the classroom setting. Prior to the survey research staff clearly explained to the students the survey procedure and the questionnaire item by item. Students were then invited to self-complete the questionnaire in the classroom. On average, the questionnaire survey took approximately 20 minutes to complete. A school teacher supervised and assisted the survey procedure in each class.

(1) Anthropometry: Students’ body weight, height and waist circumference (WC) were measured by trained research staff. Students, wearing light indoor clothing and without shoes, had their weight measured to the nearest 0.1 kilograms using a beam balance scale, and height measured to the nearest 0.01 meter using a stadiometer (Wuxi Weight Factory, Wuxi, Jiangsu, China). Using a non-elastic flexible tape, WC was measured to the nearest 0.1 centimeter at the midpoint between the costal inferior and iliac crest. Each of these anthropometric variables was measured twice and the mean of the two readings for each were used in our analysis.Overweight was defined as BMI between 85^th^ and 95^th^ percentile value, while obesity as BMI ≥ 95^th^ for age- and sex-specific reference data according to the recommendation for Chinese children by the Group of China Obesity Task Force [31]. In this Report we use the term ‘excess body weight’ (EBW) to describe being overweight or obese (BMI ≥ 85^th^ percentile value).

(2) Study variables collected in the questionnaire survey

(a) Socio-demographic characteristics: These included student’s age, gender, grade, school, ethnicity, parental educational attainment (classified into 3 subgroups: ≤9 yrs, >9 but ≤ 12 yrs, and ≥13 yrs), and family size and structure.

(b) Obesity-related knowledge: A series of questions were asked regarding knowledge about obesity and its related behavioral risk factors and long-term influence of childhood obesity. All these variables were categorized as “Yes” or “No or do not know”. For example, the question about information on screen time use was “Do you think spending prolonged time in viewing TV or using the computer can result in excess body weight?”

(c) Dietary intake: Items from a specifically validated Frequency Food Questionnaire (FFQ) were selected to gather information on dietary consumption including meat, vegetable/fruit, snack and soft-drinks [32]. All variables will be categorized into tertiles for our analysis.

(d) Physical activity and sedentary behavior: These were assessed using the Chinese version of the International Physical Activity Questionnaire (CHN-IPAQ) [33]. We will calculate moderate physical activity (MPA) time based on time spent in jogging and doubled the time in ball playing and swimming. Time spent on homework, sleep and commuting to/from school were also collected.

(e) Behavior change: Students were asked questions on changes in lifestyle and behavior (dietary intake, physical activity and sedentary behavior) at first follow-up compared to the baseline survey. Responses were recorded on a 5-point Likert scale (increasing a lot, increasing a little, no change, decreasing a little, and decreasing a lot).

### Power calculation

Power calculations were based on BMI, our primary outcome. We aimed to detect a clinically important difference of at least 0.5 kg/m^2^ between intervention and control groups, and assumed a standard deviation of 3.0 kg/m^2^. To calculate the design effect due to the cluster design of the study, we estimated the average number of participants at each school would be 150, and, based on previous research [17], that the ICC = 0.005; thus the design effect was 1.75. Consequently, we calculated that if 1200 students from 8 schools completed the study we would have 80% power to find a clinically important difference in BMI between the intervention and control groups, with α = 0.05 (one-sided).

### Statistical analysis

Descriptive statistics were calculated. Between group difference was examined for treatment assignment and overweight status. Differences were tested using independent t-tests for continuous variables (eg, BMI) and chi-square tests for categorical variables (eg, overweight, lifestyle factors and obesity related knowledge). Multilevel regression models will be used to test for post-test group differences on the main outcome variables corrected for pre-test measurements. Data will be double entered and cleaned with EpiData 3.0 (The Epidata Association, Odense, Denmark), and managed and analyzed using SPSS 13.0 (SPSS, Chicago, Illinois). P < 0.05 is used to assess statistical significance.

## Discussion

The rapid economic growth and lifestyle transition that has recently taken place in Mainland China has coincided with a remarkable rise in the prevalence of overweight and obesity among children. For example, in urban areas of coastal big cities including Nanjing City, in students aged 10–12 years old, the rate of overweight and obesity was 25.5% in boys and 14.3% in girls in 2000 [3,34]. Thus population-based obesity interventions are urgently needed in China, especially in major cities such as Nanjing. To prevent obesity it is necessary for students to maintain a good balance between energy intake and energy expenditure with sufficient consideration of children’s body growth. This can be achieved by a healthy diet and adequate daily physical activity. Our intervention aims to increase healthy eating, physical activity and to reduce sedentary behaviors among all students allocated to receive the intervention program.

Some of the social environmental factors affecting students’ energy balanced related behaviors (EBRB) in China differ from those in Western societies. For example, Chinese students have a considerably heavier study burden and spend much less time in organized sports and playing outside [23], this is mainly due to high level of competitiveness in Chinese schools. Generally, students' academic performance is the dominant measure used to assess schools' achievement by both students’ parents/guardians and local educational authorities. Consequently, students’ academic performance is the top priority for school principals and classroom teachers, especially as academic performance is closely linked to their achievement outcomes including financial incentives. In such Chinese social, cultural and economic context, any obesity prevention and other health promotion programs need to make a strong case to win schools’ and parents’ interest, support, and active involvement.

At present, most school-based obesity intervention programs have been conducted in Western countries, and so have limited generalizabiliy to the Chinese context [12-19,35]. To date very few school-based obesity intervention studies targeting a reduction in obesity/overweight rates among overall students have been conducted in Mainland China [36,37], a culturally and ethnically different society from Western communities.

This study was specifically designed to develop a feasible and effective population-based intervention program that targeted lifestyle and behavioral factors contributing to excessive weight gain in school children under the current educational context in Mainland China. There were several stages for us to undertake in the development of this study. First, based on previous data on children excess body weight and its associated risk factors in Mainland China [24-27,34,38], we realized the urgent need of developing an obesity intervention program tailored for Chinese school students. Second, we identified key environmental, lifestyle and behavior determinants for obesity in Chinese school children. Third, we thoroughly reviewed both English and Chinese literature, and identified potential intervention components. Fourth, we contacted researchers who have designed similar intervention trials. We had fruitful discussions with researchers, especially from the HEALTH-KIDS Study which was conducted in the United States of America [17]. The insights gained from these discussions were very beneficial. Fifth, we consulted responsible/key persons in local educational authorities, school principals and teachers to get their ideas and suggestions on the study. For example, all educational experts consulted believed early intervention against obesity is greatly needed in China, and strongly suggested our study would be better if it was integrated into the current educational system. Sixth, we organized an international research team for this study, including experts from US and Australia. Seventh, taking into account the Chinese educational and social system and the implications from the HEALTH-KIDS study, we developed the study protocol paying particular attention to the feasibility and long-term effect of our study in the current context of Mainland China.

Our intervention program emphasized the need for multilevel interventions, including multiple intervention components that target the classroom curriculum (both healthy dieting and physical activity), school environmental support, family involvement and fun programs/events. We integrated our intervention components into schools’ academic programs, which is critical for such health intervention programs to be acceptable and feasible in China and was really crucial for us to successfully obtain sufficient support from school administrators, teachers, and students and their parents/guardians. We implemented this study with limited impact on the schools’ academic schedule.

In summary, we designed this lifestyle intervention project with due consideration of Chinese cultural and familial tradition, social convention, and current primary education and exam system in China, and the successful experience from the Chicago-based HEALTH-KIDS obesity prevention program. We did our best to gain good support from educational authorities, school administrators, teachers and parents, and to integrate intervention components into schools’ regular academic programs. The findings and experience gained in this study will help guide future school-based childhood obesity prevention programs in China as well as to provide insight for other low- and middle income countries.

## **Competing interests**

The authors declare that they have no competing interests.

## **Author contributions**

FX (PI of the project) was responsible for study design, data collection and analysis, and manuscript writing; YW (Co-PI of the project), for study design and manuscript drafting; ZW, XH and AS, for data collection and manuscript revision; JL (Co-PI of the project), for study design, data collection and manuscript writing; RSW and LAT, for power calculating, manuscript writing and language editing. All authors read and approved the final manuscript.

## Pre-publication history

The pre-publication history for this paper can be accessed here:

http://www.biomedcentral.com/1471-2458/12/316/prepub
